# Supplementary material for: Active Force Dynamics in Red Blood Cells Under Non‐Invasive Optical Tweezers
Source: Adv Sci (Weinh). 2025 Dec 22;13(10):e14778. doi: 10.1002/advs.202514778 (PMC12915208; doi:10.1002/advs.202514778)
Supplement: Supplementary file 1 — Supporting File: advs73476‐sup‐0001‐SuppMat.docx. [file ADVS-13-e14778-s001.docx]

**Supplementary Information for**

**Active Force Dynamics in Red Blood Cells
Under Non-Invasive Optical Tweezers**

Arnau Dorn^1,2^, Clara Luque-Rioja^1,2^, Macarena Calero^2^, Diego Herráez-Aguilar^3^, Francisco Monroy^1,2, #^ and Niccolò Caselli^1,2,*^

*^1^ Departamento de Química Física, Universidad Complutense de Madrid, Ciudad Universitaria s/n, 28040 Madrid, Spain*

*^2^ Translational Biophysics, Instituto de Investigación Sanitaria Hospital Doce de Octubre, 28041 Madrid, Spain*

*^3^ Instituto de Investigaciones Biosanitarias, Universidad Francisco de Vitoria, Ctra. Pozuelo-Majadahonda, Pozuelo de Alarcón, Madrid, Spain*

**Email:** *ncaselli@ucm.es; ^#^monroy@ucm.es

**This PDF file includes:**

Supporting Information Figures S1 to S13

Supporting Information Tables T1 to T10


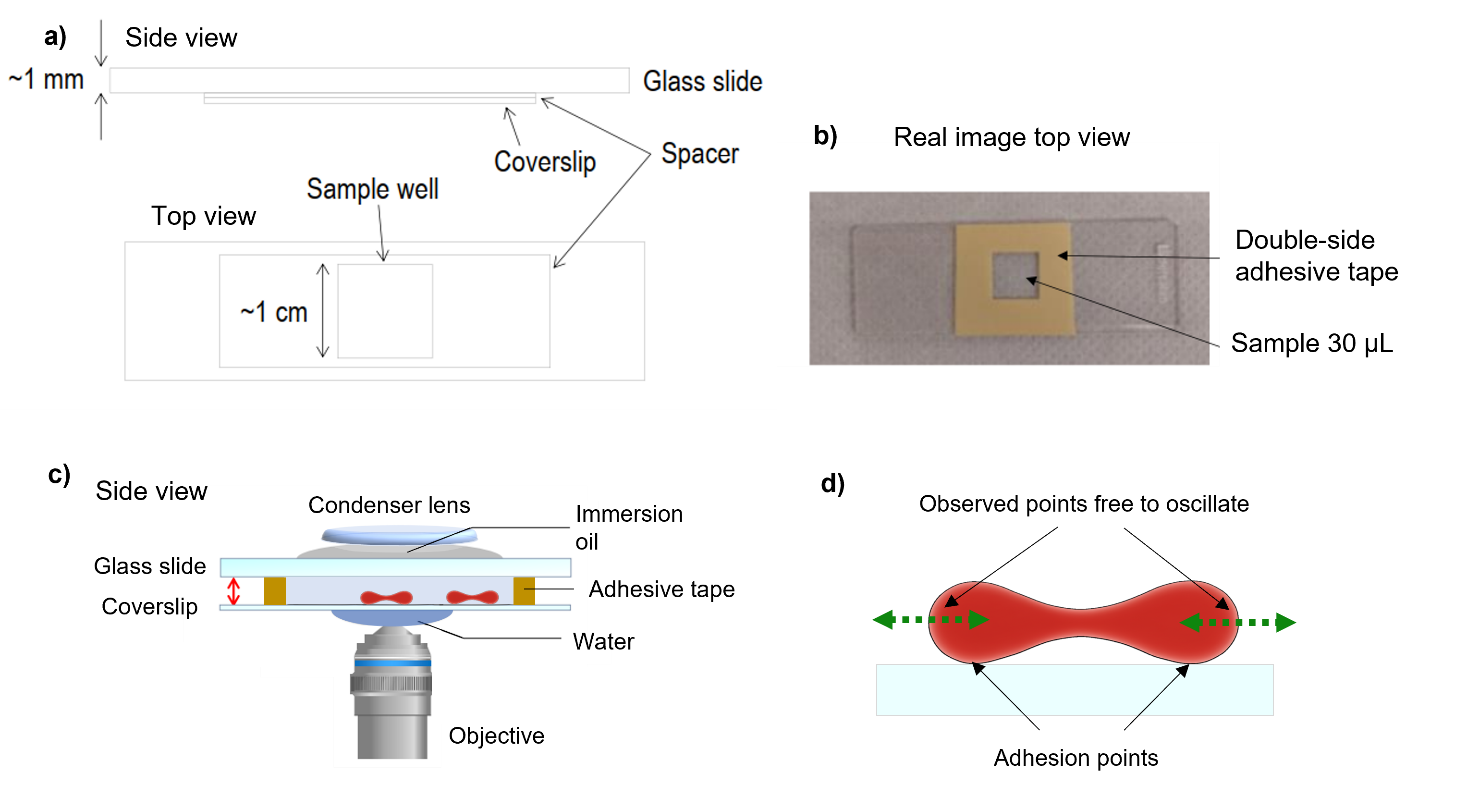


**Figure S1. Schematics of the sample chamber and mounting configuration. a)** Side and top view schematics of the sample chamber used in the experiments. **b)** Real image of the sample chamber (top view). **c)** Schematic side view (not in scale) of the experimental setup: the RBCs dilution (with discocyte shaped cells) was placed in the sample chamber under the microscope. **d)** Schematic side view of a single RBC resting on the bottom coverslip glass. Adhesion occurred locally at the bottom basal surface of the discocyte due to gravity and unspecific surface interactions. The membrane fluctuations measurements were performed in the equatorial plane of the cell, where the membrane was free to oscillate (green arrows). These positions were $\sim\mu$m distant from the adhesion points both in the equatorial plane and vertical direction. This configuration is referred to as free-standing in the main manuscript because optical traps were not applied.


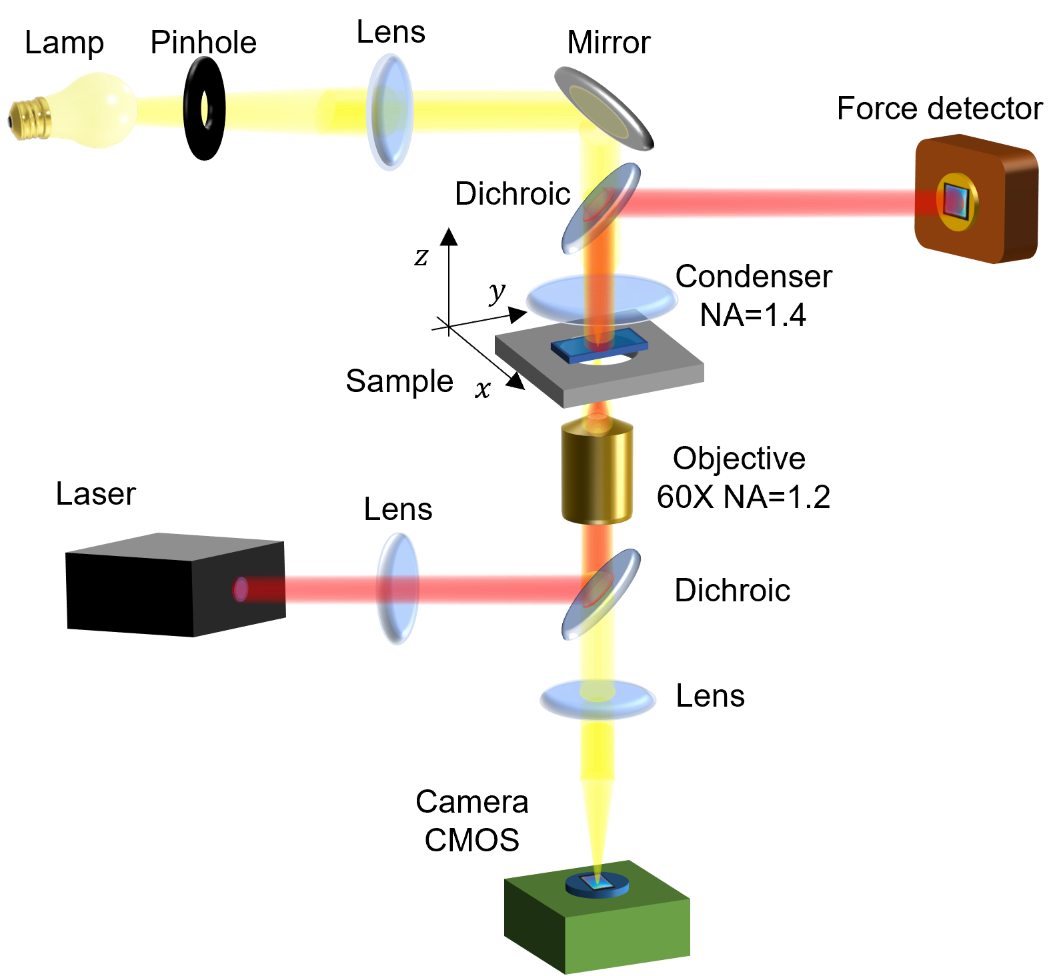


**Figure S2. Schematics of the experimental setup.** The optical path of the optical tweezers is reported as the red beam, and the optical path of bright field video-microscopy is reported as the yellow beam.


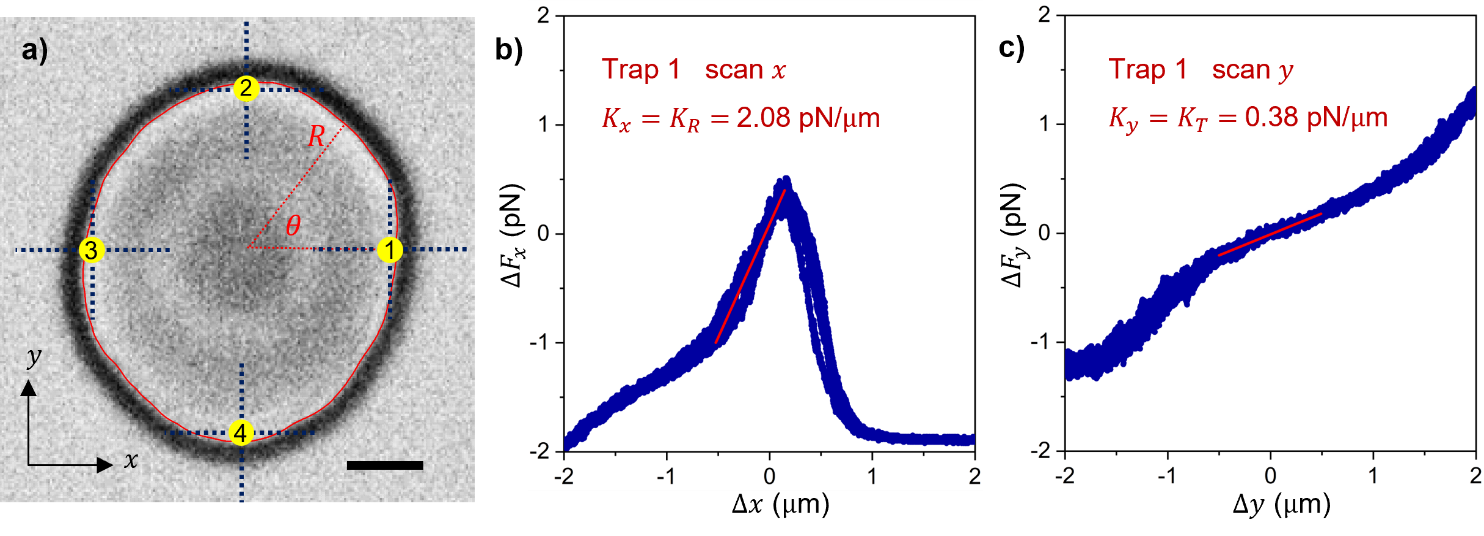
**Figure S3**. **Stiffness of optical tweezers directly applied to the RBC membrane**. **a)** Bright-field image of a healthy RBC observed in the equatorial plane $xy$. The red curve represents the membrane contour evaluated by the membrane tracking algorithm as the rim halo. $R$ and $\theta$ are the local cell radius and angle with respect to the center of the cell. They were employed to evaluate the radial displacement $\delta h\left( \theta,t \right)=R\left( \theta,t \right)-\left\langle R\left( \theta,t \right) \right\rangle$ and flickering amplitude $\sigma_{\delta h}$ along the membrane contour. The Scale bar is $2 \text{μ}$m. The four yellow dots distributed along the RBC membrane rim are the positions where single optical tweezers were applied by focusing a Gaussian laser beam with power P_1_=$1.5$ mW/trap at successive times. To retrieve the local trap stiffness, we induced a controlled displacement to each single trap by moving the laser beam. This task was accomplished by means of the SENSOCELL integrated software, which employed acousto-optic deflectors to execute the scan in few milliseconds. Dashed lines represent the trap displacements of $\pm2 \text{μ}$m in $x$ and $y$ directions. During this scanning procedure, we visually verified that the whole cell did not move globally and adhered to the glass surface, confirming that the measured force response was due to the membrane’s local resistance and the optical trap itself. **b)-c)** Optical trapping force measured when the OTs were applied at position 1, as a function of the trap displacement $\Delta x$ and $\Delta y$, respectively. The values of ∆$x = 0$, ∆𝑦 $= 0$ correspond to the intersection of the dashed lines at location 1. For this specific location, the force ${\Delta F}_{x}$ is the radial component while ${\Delta F}_{y}$ is the tangential one. Each scan was performed unidirectionally 5 times, starting from the minimum displacement of -2 $\text{μ}$m, to ensure reproducibility and all the data from those measurements were reported as scatter dots in **a)** and **b)**. The force values were reported by subtracting the initial offset force measured at the trap’s starting position (by defining ${\Delta F}_{x}=0$ at $\Delta x=0$ and ${\Delta F}_{y}=0$ at $\Delta y=0$). Red lines are the linear fit of the force data in the proximity of the initial trap position, in the range $(-0.5,+0.5)$ $\text{μ}$m. Trap stiffness was calculated as the fitting line slope in this range, assuming a harmonic trapping potential (${\Delta F}_{i}=-K_{i}{\Delta x}_{i}$). Therefore, for the trap in position 1, the stiffness calculated along $x$ corresponds to the radial contribution of the RBC membrane ($K_{x}=K_{R}$) and along $y$ to the tangential component ($K_{y}=K_{T}$). The values of radial and tangential stiffness obtained in **b)-c)** were $K_{R}=$ $2.08$ pN/$\text{μ}$m and $K_{T}= 0.38$ pN/$\text{μ}$m, respectively. The same evaluation was performed for traps placed in positions 2, 3 and 4. The radial stiffness was consistently larger than the tangential stiffness at all positions. This difference can be attributed to the fact that radial trap displacement experienced a much larger variation in the refractive index spatial distribution (when crossing the boundary between intracellular and external medium) with respect to the tangential scan, in which the laser did not reach the external medium. The different spatial variation of the refractive index resulted in a greater contribution of the optical gradient force to $K_{R}$, with respect to $K_{T}$.


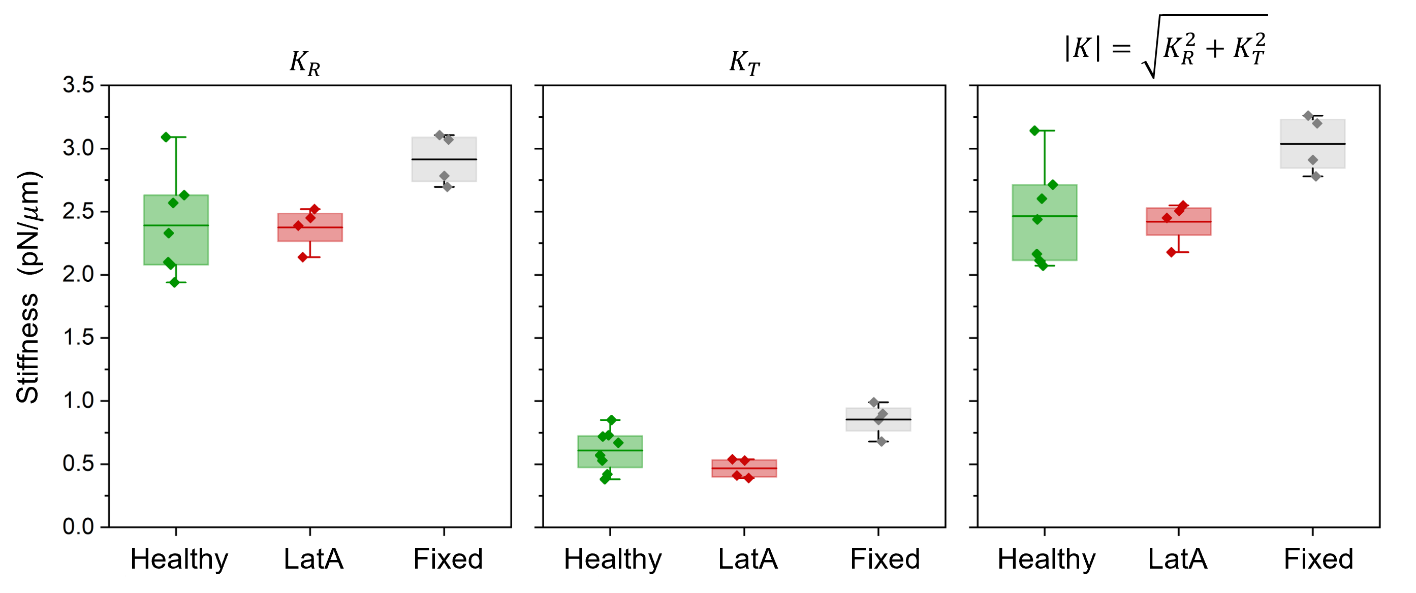


**Figure S4**. **Optical tweezers stiffness**. For each single optical trap, the stiffness was evaluated using the method described in Figure S3, employing a laser power equal to P_1_=1.5 mW/trap. $K_{T}$ represents the stiffness obtained for scans tangential to the RBC membrane. $K_{R}$ is the stiffness obtained for radial scans, normal to the RBC membrane contour. The stiffness modulus was defined as $\left| K \right|=\sqrt{K_{R}^{2}+K_{T}^{2}}$. The reported measurements were obtained for three RBCs conditions: healthy, LatA and fixed cells. Data corresponding to healthy RBCs were extracted from 4 measurements on 2 different cells (8 total data). Data for LatA-treated and Fixed RBCs were generated from 4 measurements on 1 single cell. The primary objective of these measurements was to establish the correct order of magnitude of the stiffnesses used for force detection, as the main interest in this work focuses on force fluctuations.


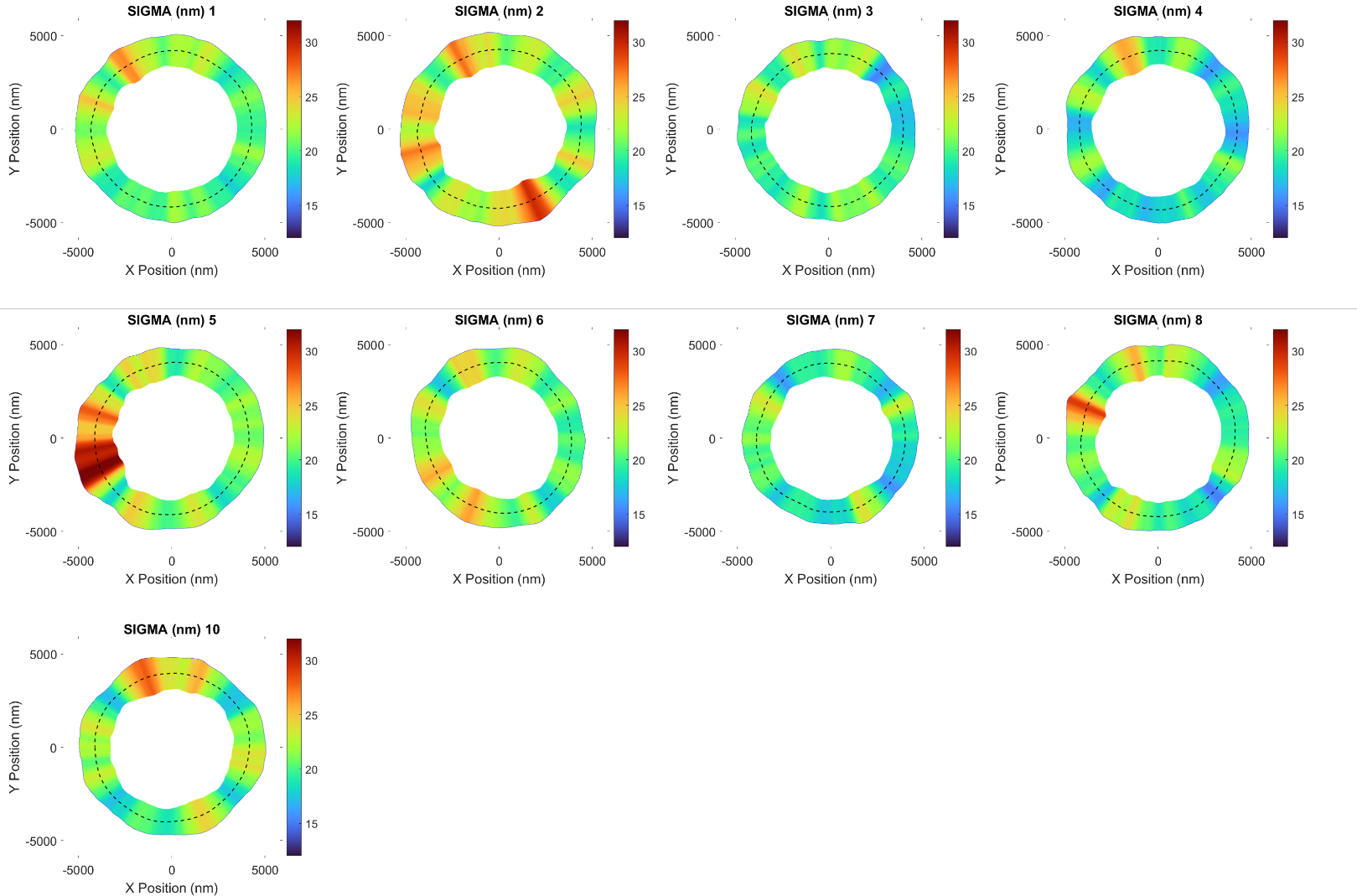


**Figure S5.** **Flickering maps for free-standing healthy RBCs**. In these measurements no optical tweezers were applied. The maps were evaluated as the standard deviation of the radial membrane fluctuation, $\sigma_{\delta h}$, and reported in the same range (12-32) nm. Dashed lines represent membrane mean positions. The XY plane coordinates have their origin coincident to the center of the RBCs. The map spatial amplitude was exaggerated and out of scale. The explanation of individual quantities and symbols here defined also applies to the following Figures S6 – S8.


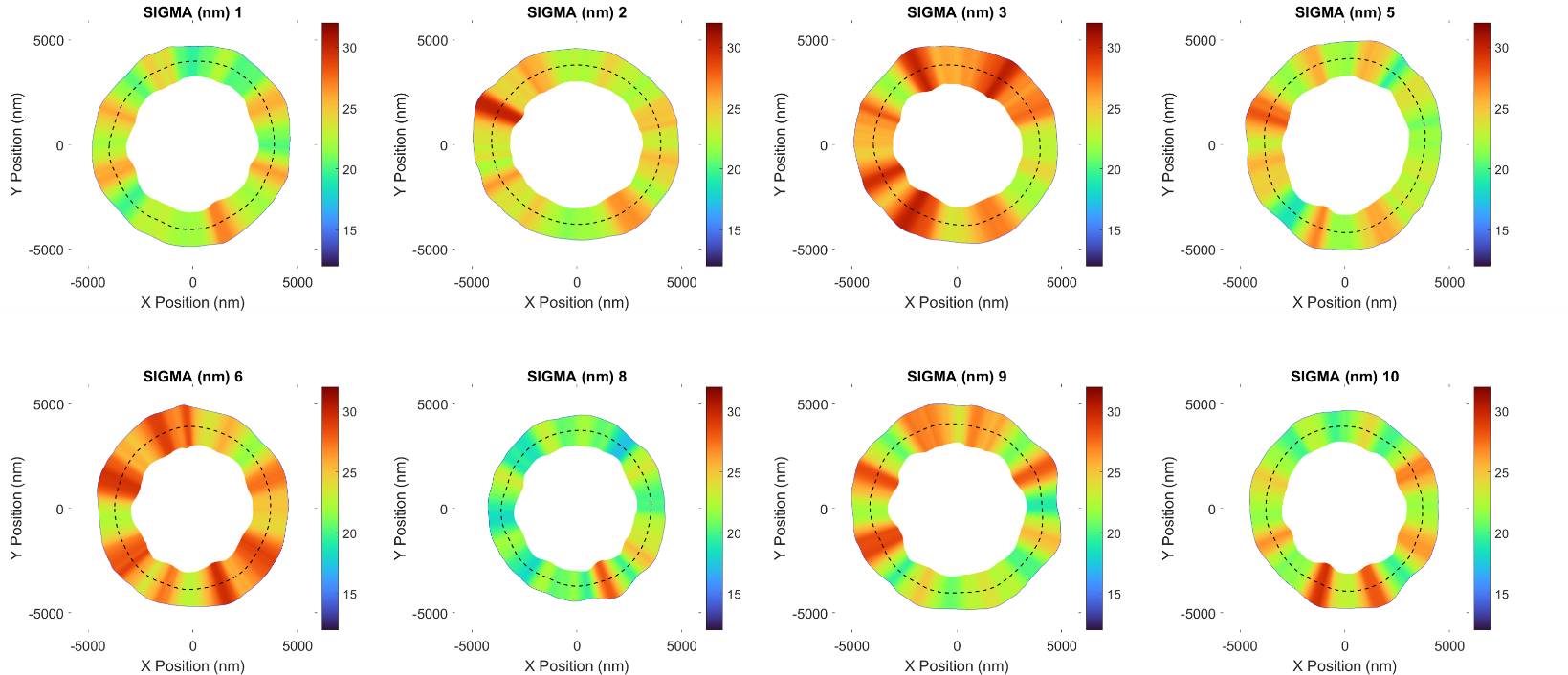


**Figure S6**. **Flickering maps for free-standing RBCs treated with Latrunculin A (LatA)**. This treatment was performed to reduce the membrane rigidity. No optical tweezers were applied during these measurements.


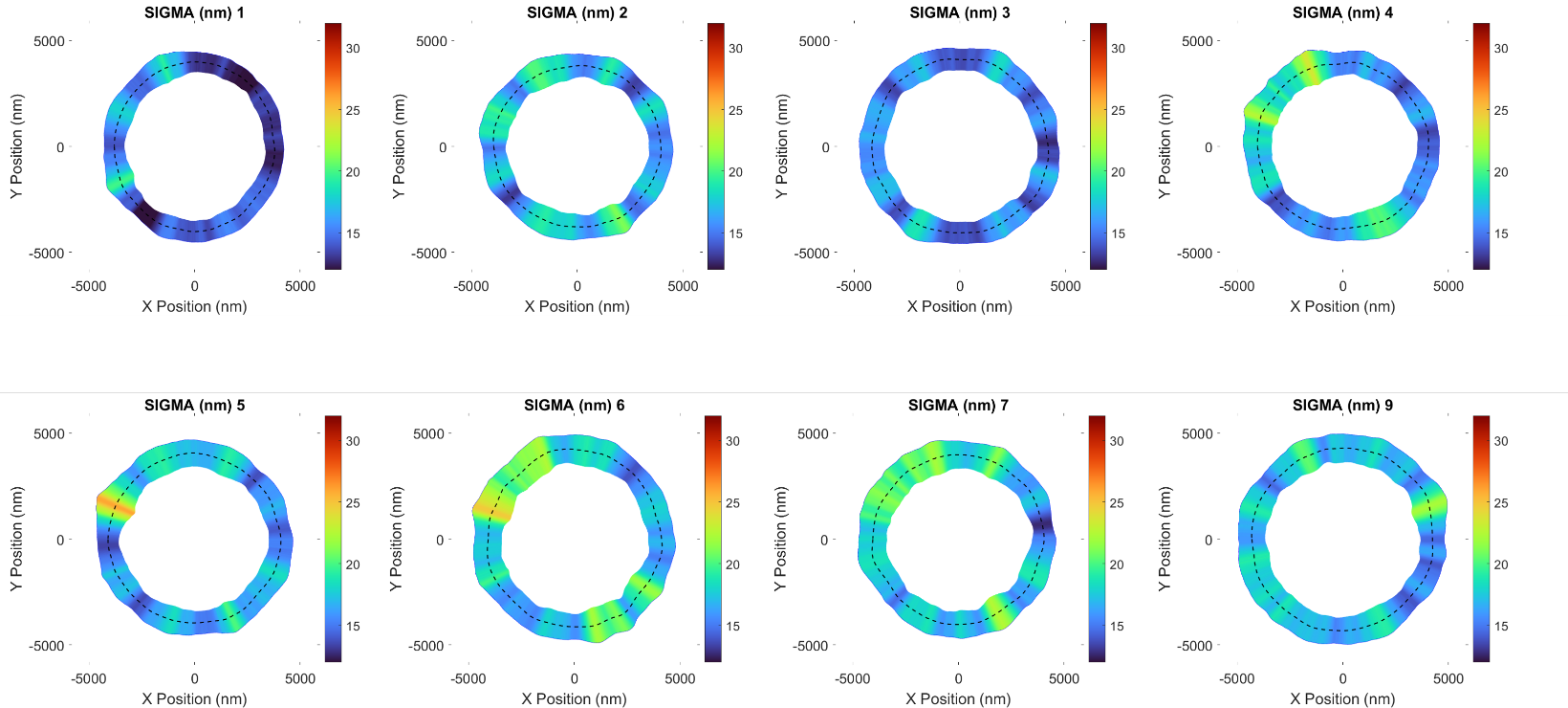


**Figure S7**. **Flickering maps for free-standing RBCs treated with inosine and iodoacetamide (ATP-)**. This treatment was performed to inhibit cytoskeletal phosphorylation and production of ATP. No optical tweezers were applied during these measurements.


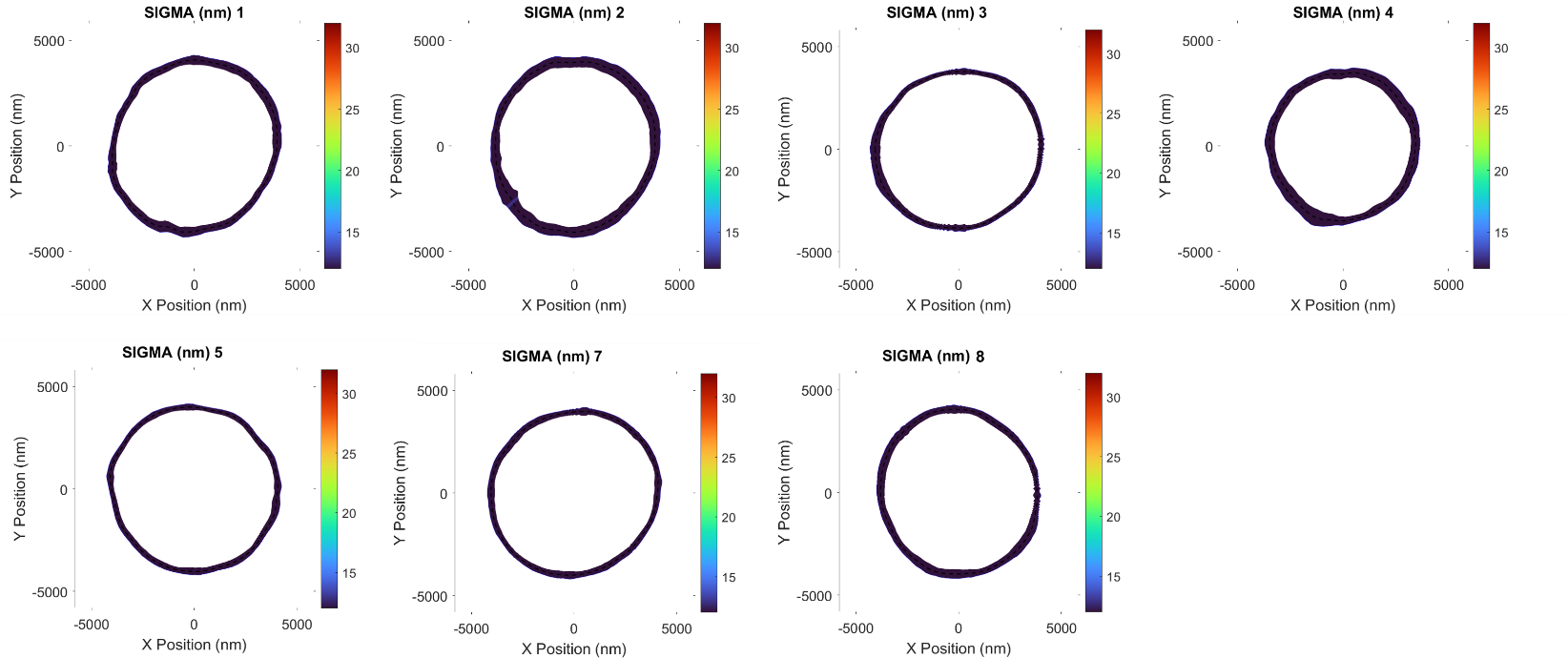
**Figure S8**. **Flickering maps for free-standing RBCs treated with glutaraldehyde (fixed)**. This treatment was performed to solidify hemoglobin and crosslink the cytoskeleton. No optical tweezers were applied during these measurements. 7 RBCs were analyzed.


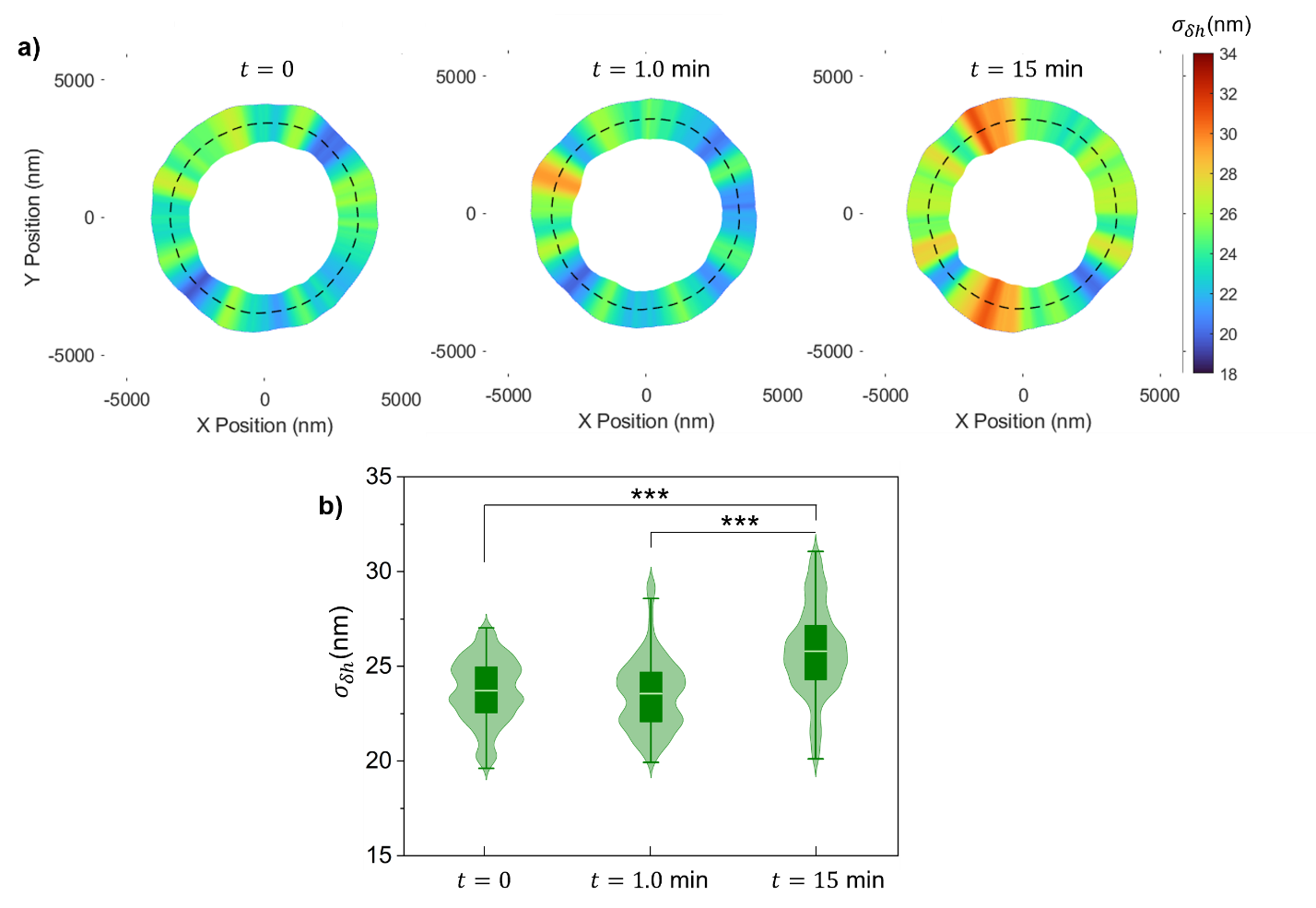


**Figure S9.** **Temporal reproducibility of membrane flickering and local hot spots. a)** Flickering maps, $\sigma_{\delta h}$, for a single healthy and free-standing RBC performed in time succession. The first map (left panel) was acquired at time $t=0$, the second at time $t=1.0$ min (central panel) and the third at time $t=15$ min (right panel). Each measurement lasted for 30 s. **b)** Distributions of $\sigma_{\delta h}$ for the data reported in a). Statistical significance of differences between distributions was found only for the measurement at the largest time point ($t=15$ min). The symbol *** indicates a statistically significant difference between the two labeled samples (pairwise two-sample $t$-test, $p\ll0.001$). The pairwise comparison shows a negligible difference between the initial and the $t=1.0$ min measurement regarding both flickering distributions and hot spots locations, thus confirming short-term persistence of flickering. Conversely, the distribution measured at time $t=15$ min shows a significant variation indicating long-term reorganization of the cytoskeletal activity.


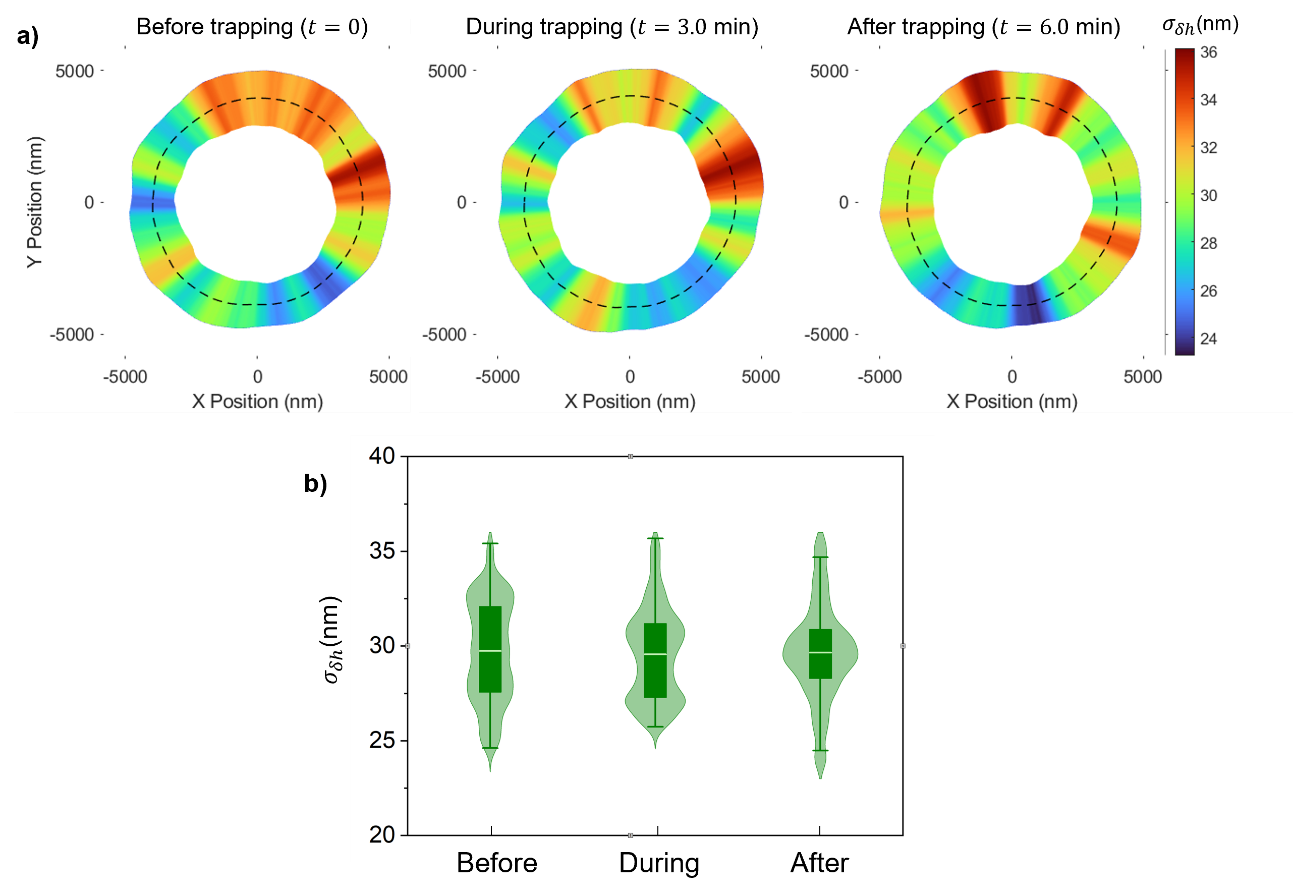


**Figure S10. Reversibility of membrane flickering after optical trapping. a)** Flickering maps, $\sigma_{\delta h}$, for a given healthy RBC performed in time succession, reported from left to right: before the application of optical trapping (free-standing cell at $t=0$, left panel), during the application of OTs at laser power P_1_ = 1.5 mW/trap (at $t=3.0$ min, central panel) and after the OTs were turned off (free-standing cell at $t=6.0$ min, right panel). Each measurement lasted for 30 s. **b)** Distributions of $\sigma_{\delta h}$ for the data reported in **a)**. No statistically significant difference was found between distributions (pairwise two-sample $t$-test showed $p>$0.05), confirming that the mechanical effects induced by the optical traps were reversible and possibly did not damage the cell structure. However, after 6 min the local hot spots were redistributed along the membrane contour. The RBC presented in this Figure originated from a different healthy donor than the one used for the cells studied in the main manuscript.


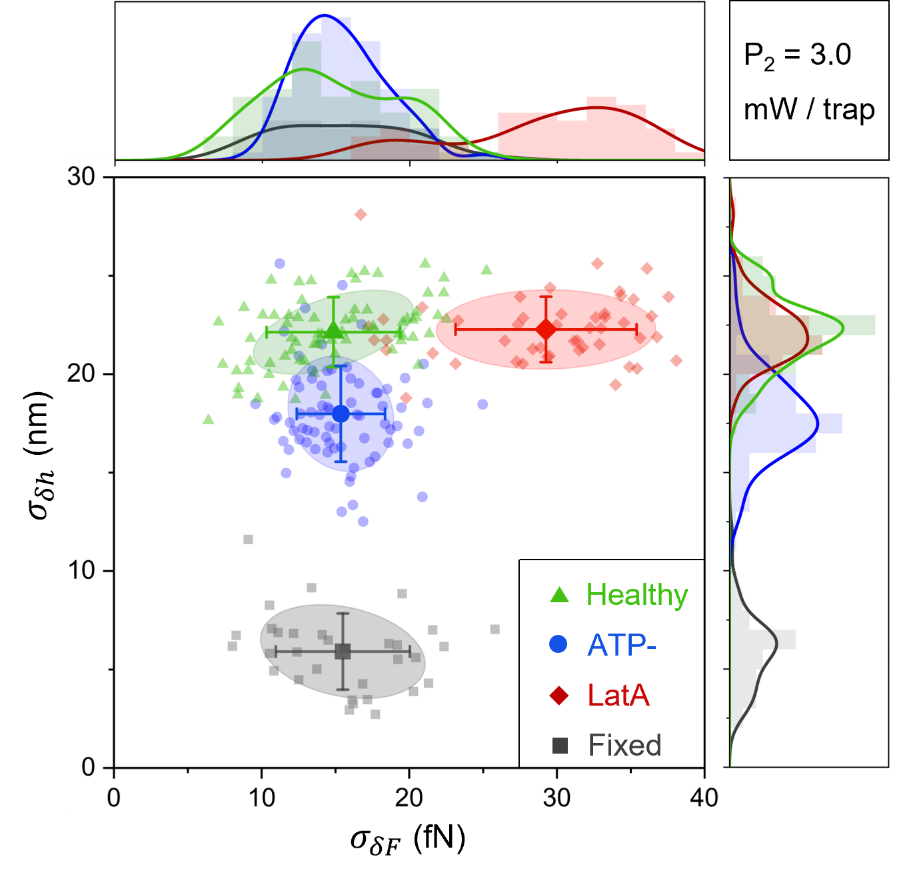


**Figure S11**. **Dynamics map for RBCs probed with optical tweezers at laser power P_2_=3.0 mW/trap.** The vertical axis reports the standard deviation of the local membrane radial fluctuation, $\sigma_{\delta h}$, and the horizonal axis the standard deviation of the radial component of the force variation, $\sigma_{\delta F}$, measured by optical tweezers applied at the same membrane position. Different groups correspond to healthy RBCs (green triangles); RBCs treated with Latrunculin A (LatA, red diamonds); RBCs treated with inosine and iodoacetamide (ATP-, blue circles); RBCs treated with Glutaraldehyde (Fixed, gray squares). The bigger symbol in each ensemble is the mean value, error bars are the standard deviation of the group distribution. Ellipses correspond to the 70% confidence regions. The top and right distributions are the projections of the dynamics map on the $\sigma_{\delta F}$ and $\sigma_{\delta h}$ axis, respectively. Data are reported for OTs at laser power equal to P_2_=3.0 mW/trap and were extracted form $N=$10 RBCs for the healthy, LatA, and ATP- samples, and $N=$7 RBCs for the fixed sample.


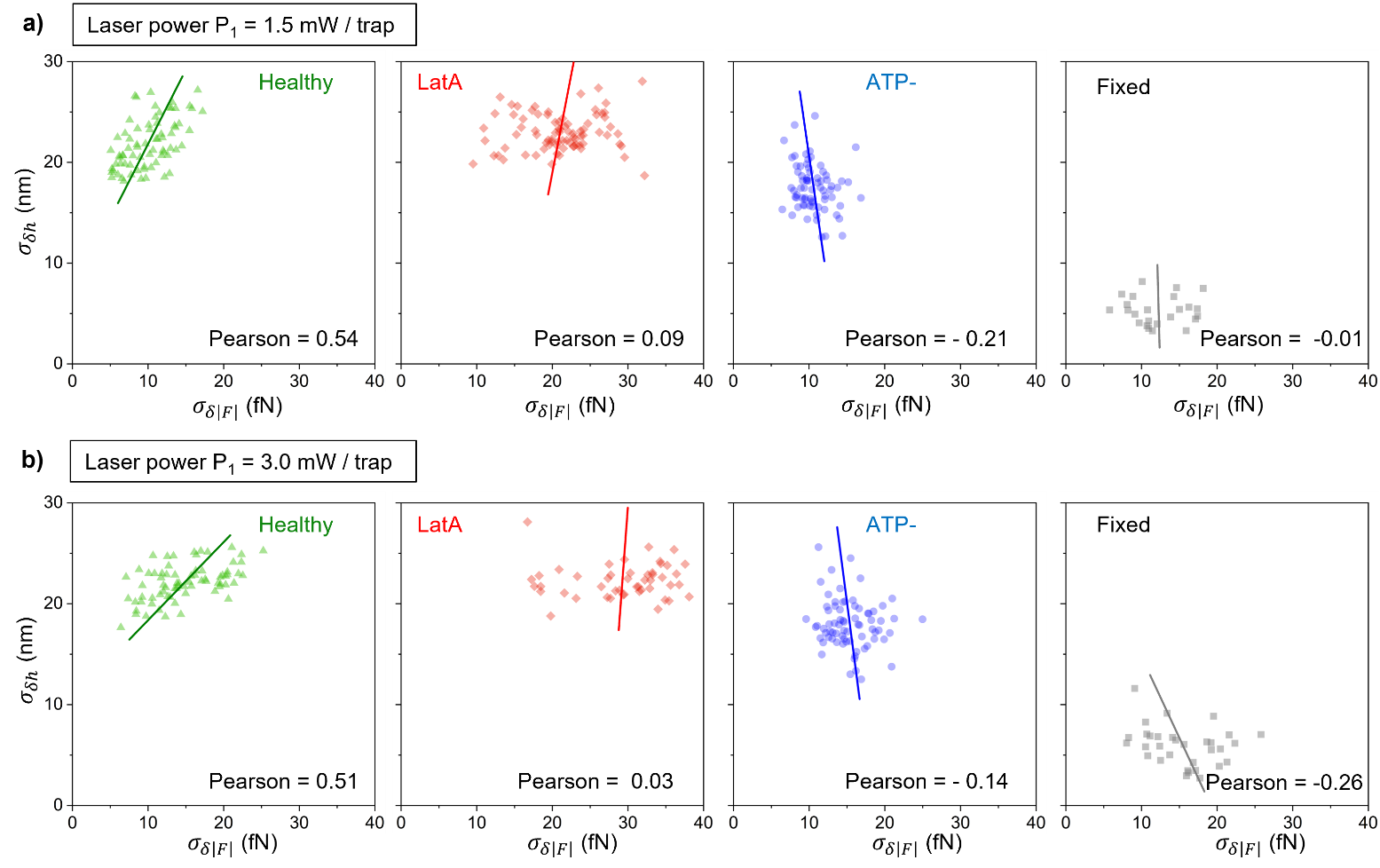


**Figure S12. Dynamics maps correlation analysis.** Data reported in Figure 5 and S11 were reported in single panels separating them for sample and laser power. The $\sigma_{\delta h}$ - $\sigma_{\delta F}$ space was reported for RBCs probed by optical tweezers at laser power P_1_=1.5 mW/trap **(a)**, and P_2_=3.0 mW/trap **(b)**. The Pearson linear correlation coefficient was reported for each case and the liner fit of the data was represented as a straight line in each panel.


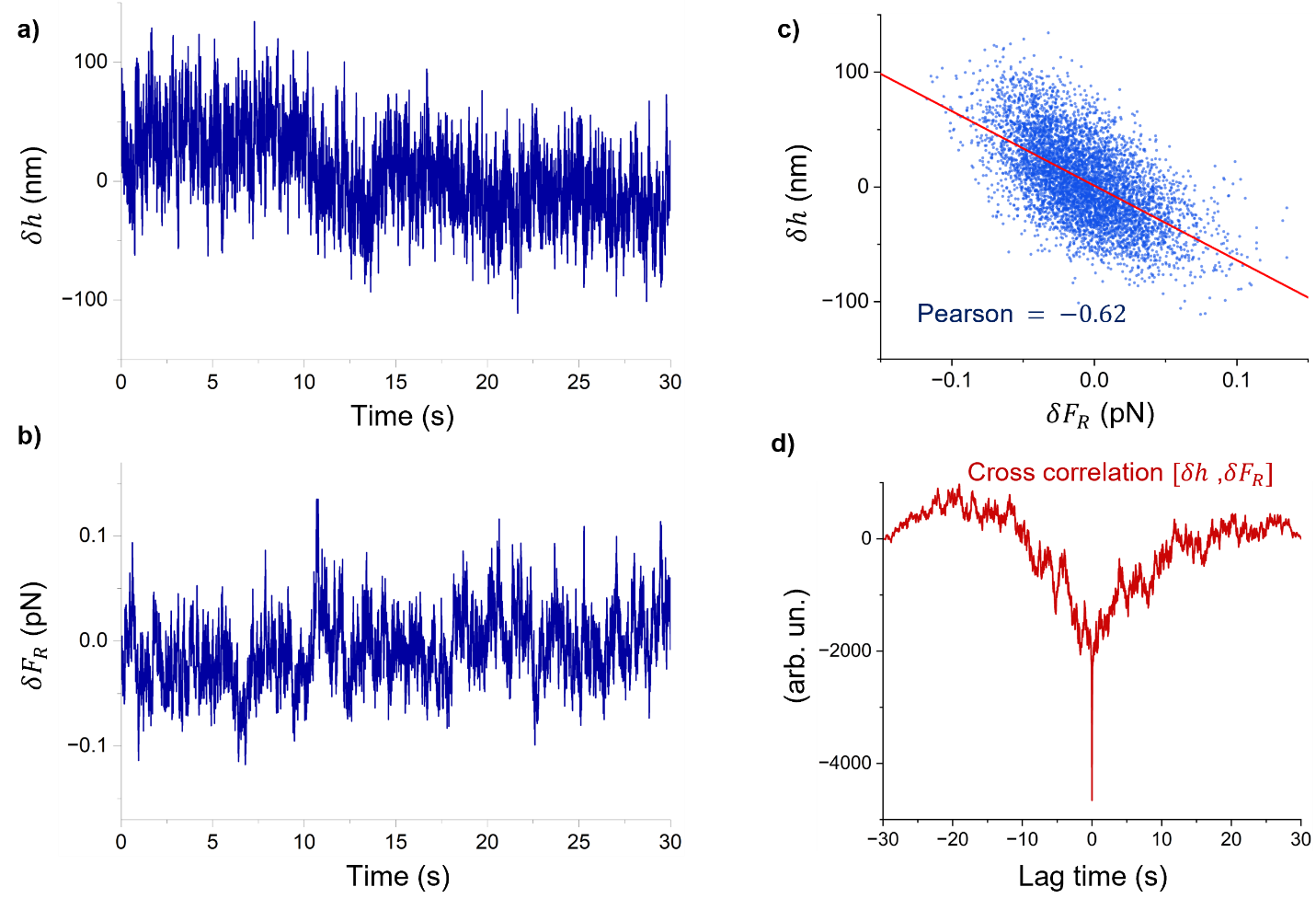
**Figure S13. Correlation between force and spatial fluctuation at a given membrane position. a)-b)** Time series of spatial fluctuations, $\delta h(t)$, and radial force fluctuations, $\delta F_{R}(t)$, respectively. Both signals were detected at a given trap position on the membrane of a healthy RBC at laser power P_1_=1.5 mW/trap. Both time series were plotted with a frame rate of 65 Hz, by averaging the force data, as discussed in Methods. **c)** Data presented in a) and b) were represented in the $[\delta h$, $\delta F_{R}]$ space. Echa data point corresponds to a given acquisition time. The red line is the linear fit of the data and represent a guide to the eye for highlighting the distribution trend. The Pearson linear correlation coefficient was evaluated as equal to $-0.62$, indicating a high level of anti-correlation between the two independently measured variables, in agreement with the harmonic approximation $\delta F_{R}=-K\delta h$. **d)** Cross correlation between the two time-series $\delta h(t)$, $\delta F_{R}(t)$ evaluated as a function of the lag time by using the internal correlation function in the software *OrginPro 2023*. The symmetric cross correlation exhibited a negative minimum at zero lag time, indicating a high level of anticorrelation between the two variables. The same results indicating an anticorrelation were found for different trap positions and RBCs.

**Table T1. Stiffness Values of** $\boldsymbol{K}_{\boldsymbol{R}}$**,** $\boldsymbol{K}_{\boldsymbol{T}}$ **and** $\left| \boldsymbol{K} \right|$ **for each RBCs sample.** All stiffnesses are visualized in Figure S4 were reported as the mean $\pm$ standard deviation. No significant differences were observed in any component between healthy and LatA-treated cells, indicating the treatment did not affect overall membrane stiffness. In contrast, fixed cells showed significantly higher stiffness values, approximately a 20% increase in $\left| K \right|$, possibly due to induced protein crosslinking. Despite these stiffness differences, the biconcave shape of the cells remained constant throughout the experiments, with the appearance of less than 10% echinocytes in every sample by the end of the measurement session (approximately 3-4 hours after sample preparation).

|  | $K_{R}$ (pN/$\text{μ}$m) | $K_{T}$ (pN/$\text{μ}$m) | $\left\vert K \right\vert$ (pN/$\text{μ}$m) |
| --- | --- | --- | --- |
| Healthy RBCs | $2.4\pm0.4$ | $0.61\pm0.16$ | $2.5\pm0.4$ |
| LatA RBCs | $2.38\pm0.18$ | $0.47\pm0.08$ | $2.4\pm0.2$ |
| Fixed RBCs | $2.9\pm0.2$ | $0.86\pm0.16$ | $3.0\pm0.2$ |

**Table T2**. Analysis of the data reported in Figure 2 c) of the main manuscript. Second column: average value of $\sigma_{\delta h}$, with uncertainty given by its standard deviation, evaluated for each sample. Right box: probability ($p$-value) calculated from all the pairwise $t$-tests.

|  | $\sigma_{\delta h}$ (nm) |  | $p$-value | | | |
| --- | --- | --- | --- | --- | --- | --- |
|  |  |  | Healthy | LatA | ATP- | Fixed |
| Healthy | $21.0\pm2.5$ |  |  |  |  |  |
| LatA | $23.8\pm2.5$ |  | ${10}^{-63}$ |  |  |  |
| ATP- | $16.7\pm2.2$ |  | ${<10}^{-100}$ | ${<10}^{-100}$ |  |  |
| Fixed | $6.8\pm1.5$ |  | ${<10}^{-100}$ | ${<10}^{-100}$ | ${<10}^{-100}$ |  |

**Table T3**. Analysis of the data reported in Figure 3 c) of the main manuscript for healthy RBCs considering all tracked membrane positions (green data). Second column: average value of $\sigma_{\delta h}$, with uncertainty given by its standard deviation, evaluated for each sample at different trapping laser power. Right box: probability ($p$-value) calculated from every pairwise $t$-test.

|  | $\sigma_{\delta h}$ (nm) | $p$-value | | |
| --- | --- | --- | --- | --- |
|  |  | P_0_ | P_1_ | P_2_ |
| P_0_ | $21.0\pm2.5$ |  |  |  |
| P_1_ | $21.2\pm2.4$ | $0.41$ |  |  |
| P_2_ | $21.5\pm2.2$ | ${3\times10}^{-3}$ | ${7\times10}^{-3}$ |  |

**Table T4**. Analysis of the data reported in Figure 3 c) of the main manuscript for healthy RBCs considering only membrane positions where optical traps were applied (orange data). Second column: average value of $\sigma_{\delta h}$, with uncertainty given by its standard deviation, evaluated for each sample at different trapping laser power P_1_ and P_2_. Right box: probability ($p$-value) calculated from pairwise $t$-test between $\sigma_{\delta h}$ evaluated for all membrane positions and $\sigma_{\delta h}$ only at positions where optical traps were generated, at laser power P_1_ and P_2_, respectively.

|  |  | $\sigma_{\delta h}$ (nm) | $p$-value ($\sigma_{\delta h}$ all positions vs traps positions) | |
| --- | --- | --- | --- | --- |
| P_1_ (traps) |  | $21.9\pm2.5$ | P_1_ | ${3\times10}^{-2}$ |
| P_2_ (traps) |  | $22.2\pm2.0$ | P_2_ | ${5\times10}^{-3}$ |

**Table T5**. Analysis of the data reported in Figure 4 of the main manuscript for RBCs considering all tracked membrane positions under LatA (red data) and ATP- (blue data) treatments. Average value of $\sigma_{\delta h}$, with uncertainty given by its standard deviation, evaluated for each sample at different trapping laser power and the probability ($p$-value) calculated from every pairwise $t$-test.

| LatA | $\sigma_{\delta h}$ (nm) | $p$-value | | |
| --- | --- | --- | --- | --- |
|  |  | P_0_ | P_1_ | P_2_ |
| P_0_ | $23.8\pm2.5$ |  |  |  |
| P_1_ | $21.9\pm2.1$ | ${10}^{-39}$ |  |  |
| P_2_ | $21.8\pm1.7$ | ${10}^{-35}$ | $0.37$ |  |

| ATP- | $\sigma_{\delta h}$ (nm) | $p$-value | | |
| --- | --- | --- | --- | --- |
|  |  | P_0_ | P_1_ | P_2_ |
| P_0_ | $16.7\pm2.2$ |  |  |  |
| P_1_ | $16.9\pm2.2$ | $0.22$ |  |  |
| P_2_ | $17.5\pm2.4$ | ${10}^{-7}$ | ${10}^{-5}$ |  |

**Table T6**. Analysis of the data reported in Figure 4 of the main manuscript for LatA and ATP- treated RBCs considering only membrane positions where optical traps were applied (orange data). In the third column are reported the average value of $\sigma_{\delta h}$, with uncertainty given by its standard deviation, evaluated for each sample at different trapping laser power P_1_ and P_2_. In the right box are shown the probabilities ($p$-value) calculated from pairwise $t$-test between $\sigma_{\delta h}$ evaluated for all membrane positions and $\sigma_{\delta h}$ only at positions where optical traps were generated, for both samples at laser power P_1_ and P_2_, respectively.

|  |  | $\sigma_{\delta h}$ (nm) | $p$-value ($\sigma_{\delta h}$ all positions vs traps positions) | |
| --- | --- | --- | --- | --- |
| LatA | P_1_ (traps) | $23.1\pm1.8$ | P_1_ | ${4\times10}^{-6}$ |
|  | P_2_ (traps) | $22.6\pm1.7$ | P_2_ | ${9\times10}^{-3}$ |
| ATP- | P_1_ (traps) | $17.7\pm2.4$ | P_1_ | ${3\times10}^{-3}$ |
|  | P_2_ (traps) | $18.2\pm2.5$ | P_2_ | ${2\times10}^{-2}$ |

**Table T7**. Average value of $\sigma_{\delta h}$ and $\sigma_{\delta F}$, with uncertainty given by its standard deviation, evaluated for each sample at laser power P_1_ and P_2_, corresponding to the force data reported in in the dynamic maps of Figure 5 of the main manuscript and Figure S11 of the Supplementary information, respectively.

|  | Power P_1_ | | Power P_2_ | |
| --- | --- | --- | --- | --- |
|  | $\sigma_{\delta h}$ (nm) | $\sigma_{\delta F}$ (fN) | $\sigma_{\delta h}$ (nm) | $\sigma_{\delta F}$ (fN) |
| Healthy | $21.9\pm2.5$ | $10\pm3$ | $22.1\pm1.8$ | $15\pm5$ |
| LatA | $23.0\pm1.8$ | $21\pm5$ | $22.3\pm1.7$ | $29\pm6$ |
| ATP- | $17.5\pm2.4$ | $11\pm2$ | $18.0\pm2.4$ | $15\pm3$ |
| Fixed | $5.3\pm1.4$ | $12\pm4$ | $5.9\pm1.9$ | $16\pm5$ |

**Table T8**. **Statistical analysis of the force data reported in Figure 5.** Second column: average value of $\sigma_{\delta F}$, with uncertainty given by its standard deviation, evaluated for each sample under optical trapping at laser power P_1_. Right box: probability ($p$-value) calculated from all the pairwise $t$-tests.

|  | $\sigma_{\delta F}$ (fN) |  | $p$-value | | | |
| --- | --- | --- | --- | --- | --- | --- |
|  |  |  | Healthy | LatA | ATP- | Fixed |
| Healthy | $10\pm3$ |  |  |  |  |  |
| LatA | $21\pm5$ |  | ${<10}^{-33}$ |  |  |  |
| ATP- | $11\pm2$ |  | $0.21$ | ${<10}^{-100}$ |  |  |
| Fixed | $12\pm4$ |  | $0.004$ | ${<10}^{-34}$ | $0.008$ |  |

**Table T9**. Analysis of the data reported in Figure 6 b) of the main manuscript. Second column: average value of the dissipated power, with uncertainty given by its standard deviation, evaluated for each sample. Right panel: probability ($p$-value) calculated from all the pairwise $t$-tests of the power distributions.

|  | Power (k_B_T/s) | $p$-value | | | |
| --- | --- | --- | --- | --- | --- |
|  |  | Healthy | LatA | ATP- | Fixed |
| Healthy | $18.1\pm6.1$ |  |  |  |  |
| LatA | $24.7\pm8.5$ | ${10}^{-7}$ |  |  |  |
| ATP- | $12.9\pm6.3$ | ${10}^{-5}$ | ${10}^{-14}$ |  |  |
| Fixed | $5.3\pm2.9$ | ${10}^{-14}$ | ${10}^{-15}$ | ${10}^{-7}$ |  |

**Table T10. Flickering variability of RBCs from 5 different healthy donors.** The experimental conditions for the controls were 25 °C. Values of $\sigma_{\delta h}$ represent mean ± SD across N=10 cells per donor. The flickering amplitude $\sigma_{\delta h}$ values were consistent across donors, with no systematic dependence on sex, age, or blood type. Inter-donor variability was found within ~10%, confirming reproducibility across healthy individuals. The donor listed as Control 4 in the table was used as the source of RBCs analyzed in the main manuscript.

| **Control ID** | **Sex / Age (years)** | **Blood serotype** | $\boldsymbol{\sigma}_{\boldsymbol{\delta h}}$ **(nm)** | **N (cells)** |  |
| --- | --- | --- | --- | --- | --- |
| Control 1 | Female / 17 | O− | 24 ± 4 | 10 | |
| Control 2 | Male / 16 | A+ | 23 ± 3 | 10 | |
| Control 3 | Female / 25 | B− | 25 ± 4 | 10 | |
| Control 4 | Male / 23 | O+ | 21 ± 3 | 10 | |
| Control 5 | Male / 45 | O+ | 22 ± 5 | 10 | |
